# Dorsal and ventral fronto-amygdala networks underlie risky decision-making in age-related cognitive decline

**DOI:** 10.1007/s11357-023-00922-2

**Published:** 2023-09-12

**Authors:** Ping Ren, Manxiu Ma, Yuchuan Zhuang, Jiayin Huang, Meiling Tan, Donghui Wu, Guozhi Luo

**Affiliations:** 1 Department of Geriatric Psychiatry, Shenzhen Mental Health Center/Shenzhen Kangning Hospital, Shenzhen, Guangdong China; 2 Queensland Brain Institute, University of Queensland, St. Lucia, QLD Australia; 3 Department of Electrical and Computer Engineering, University of Rochester, Rochester, NY USA

**Keywords:** Risky decision-making, Aging, Fronto-amygdala network, fMRI, Cognitive decline

## Abstract

Older adults often have difficulty in making decisions under uncertainty, increasing the risk of financial exploitation. However, it is still under investigation about the extent to which cognitive decline influences risky decision-making and the underlying neural correlates. We hypothesized that the individual differences of risk-taking behavior depend on cognitive integrity, in which the dorsal and ventral fronto-amygdala connectivity would play dissociable roles. In the current study, thirty-six young and 51 older adults were tested with the Iowa gambling task combing resting-state and task-related functional magnetic resonance imaging. The results showed significant changes in behaviors and the fronto-amygdala network in older adults relative to young adults. More importantly, age-effect on risk-taking behaviors was remarkably different in cognitively normal and impaired older adults. In resting-state analysis, task performance was positively correlated with the ventral fronto-amygdala connectivity and negatively correlated with the dorsal fronto-amygdala connectivity in cognitively impaired older adults, compared with cognitively normal individuals. Furthermore, task-related analysis confirmed the relationships between dorsal/ventral fronto-amygdala network and risk-taking behaviors depending on cognitive integrity. These findings indicate that the fronto-amygdala network is crucial for understanding altered risky decision-making in aging, suggesting dissociable contributions of the dorsal and ventral pathways in the context of cognitive decline.

**Supplementary Information:**

The online version contains supplementary material available at 10.1007/s11357-023-00922-2.

## Introduction

Decision making under uncertainty, as an essential component of everyday life, has been widely investigated in psychological, neuroimaging and neuropsychological studies [1–4]. Individuals need to maximize their benefits by estimating potential gains and losses in volatile environments. Due to age-related brain deterioration, older adults often have difficulty in making decisions under uncertainty, increasing the risk of financial exploitation. Previous studies have reported that older individuals could not differentiate risky and safe choices, failing to avoid penalties and generate optimal strategies [5, 6]. It has been hypothesized that age-induced decision-making deficits are associated with the degradation of the frontal cortex [7, 8] and the subcortical regions involved in the dopaminergic system [5, 9]. However, the existing evidence in the literature is controversial, and whether risky decision-making naturally declines with aging is still under debate.

Reward-based learning tasks such as the Iowa gambling task (IGT) have been widely used to investigate individual’s decision process under uncertainty, in which probabilities and possible outcomes need to be learned through feedback from previous choices [10]. Previous studies have provided converging evidence for the test–retest reliability and validity of the IGT in assessing decision making in both healthy and clinical samples [11–14]. Compared with other decision-making tasks such as the balloon analogue risk task and Columbia card task, the IGT is considered to be a better clinical instrument for assessing decision-making process [15, 16]. The inability to make optimal choices in a complex decision-making situation such as IGT, has been found in normal aging and dementia. Compared with young adults, a large portion of older adults showed impaired IGT performance defined by advantageous versus disadvantageous selections [17, 18]. However, other study showed comparable performance in the two populations [19]. A recent study reported no significant change of IGT performance in mild cognitive impairment (MCI) relative to normal aging [1]. These confounding results suggest large individual differences of risk-taking behavior within the elderly population. We speculated that risky decision-making may not be monotonically changed in natural aging, and large individual differences in risk behavior might depend on cognitive integrity.

Blood oxygen level-dependent (BOLD) functional magnetic resonance imaging (fMRI) is a promising tool for functional brain mapping, which can provide brain activation patterns related to specific cognitive tasks. Over the past decades, task-related fMRI studies have identified BOLD activations associated with IGT performance in multiple cortical and subcortical brain regions, including the middle frontal cortex (MFC), orbitofrontal cortex (OFC), ventral striatum and amygdala [10, 20]. As a core hub in subcortical regions, the amygdala strongly project to the prefrontal cortex (PFC) and also receive substantial projections from the PFC [21, 22]. The bidirectional communications between the amygdala and the PFC have been found in modulating multiple complex behaviors, such as decision making and social activity [22, 23]. For instance, previous study has reported that the medial PFC-amygdala functional connectivity was positively associated with risk-tolerance in value-based decision making task [3]. Lesions of the amygdala or ventromedial prefrontal cortex comprising the OFC have been found associated with poor performance in the IGT [24, 25]. Consistently, animal studies have found that the OFC-amygdala disconnection leads to disability to recalibrate cost–benefit decision-making during the IGT [26]. In recent studies, older adults have been found remarkable changes in the fronto-amygdala connectivity in emotion regulation [27], and self-perceived control [28]. However, it is still unclear how the amygdala interacts with the frontal subregions in modulating risky decision-making, especially in older adults.

Notably, the dorsal and ventral frontal regions may contribute differently in reward-based decision making. Using a conditioning approach–avoidance task, researchers found that the dorsal and ventral frontal signals showed opposite correlation patterns in response to increased threat [29]. Lesion of the medial orbitofrontal cortex (MOFC) significantly reduces risk-seeking behaviors [30], while lesion of the dorsolateral PFC (DLPFC) leads to a general diminished sensitivity to risk [31]. A model-driven fMRI study reported that the ventromedial and dorsolateral PFC compared costs and benefits by computing the difference between neural signatures of anticipated benefits and costs derived from the ventral striatum and amygdala, respectively [32]. Therefore, we postulated that the dorsal and ventral frontal regions may interact with the amygdala differently to regulate risk-taking behavior, and impairments of these neural circuits would lead to altered decision strategy in aging.

In the present study, we investigated the fronto-amygdala functional network in modulating risk-taking behaviors in older adults with different severity of cognitive decline. To address this, a customized IGT was used to assess risk-taking strategy and underlying neural correlates, in conjunction with resting-state and task-related fMRI methods. Although a number of neuroimaging studies have examined task-related brain activity during the IGT [10, 20, 33], resting-state fMRI allows the study of brain networks in the absence of explicit tasks which would provide significant advantages in understanding age-related alterations in decision making under uncertainty. To our knowledge, there have been limited researches examining the relationships between IGT performance and functional connectivity/regional activity during the resting state [13, 34], and only one study examined the bran networks (i.e., default mode network and fronto-parietal network) in both resting-state and IGT-related scans [35]. Therefore, the current study combined resting-state and task-related fMRI data to reveal the connectivity and brain activation within the fronto-amygdala network. We hypothesized that cognitive decline may not disrupt risky decision-making in elderly people directly, but change the neural correlates underlying risk-taking behaviors. Furthermore, we expected that the dorsal and ventral fronto-amygdala networks would contribute differently in regulating risky decision-making in the context of cognitive decline.

## Material and methods

### Participants

Thirty-eight young adults (aged 20–27 years) and 60 older adults (aged 56–80) were recruited from multiple communities in Shenzhen. All participants were right-handed determined by the Edinburgh handedness inventory, have adequate visual and auditory acuity for testing by self-report. The individuals with one of the following conditions were excluded: (1) a history of diagnosed neurological or psychiatric diseases (i.e., major depression, anxiety, or cerebrovascular disease); (2) MRI contraindications (i.e., metallic implant, claustrophobia, or pacemaker). Each participant was required to sign a written informed consent form after a full written and verbal explanation of the study. This study was performed in accordance with the Declaration of Helsinki and had been approved by the Ethics Committee of Shenzhen Kangning Hospital.

For each participant, individual’s global cognitive function was assessed using the Mini-mental State Examination (MMSE) and Montreal Cognitive Assessment (MoCA). The MMSE is a 30-point questionnaire that is widely used in clinical and research settings to assess cognitive function in multiple domains, including orientation, short-term memory, spatial abilities and so on [36]. The MoCA is a widely used screening tool for assessing global cognitive ability, showing high sensitivity in detecting cognitive impairment [37, 38]. In order to examine the effect of cognitive decline on risky decision-making, the current analysis included older adults with an extensive range of MoCA (20–30) and MMSE (16–29), and individuals with lower scores of neurocognitive assessments were not excluded. Therefore, it allowed for testing risk-taking behaviors and corresponding neural correlates in cognitively normal and declined older adults.

### Iowa gambling task

**Fig. 1:**
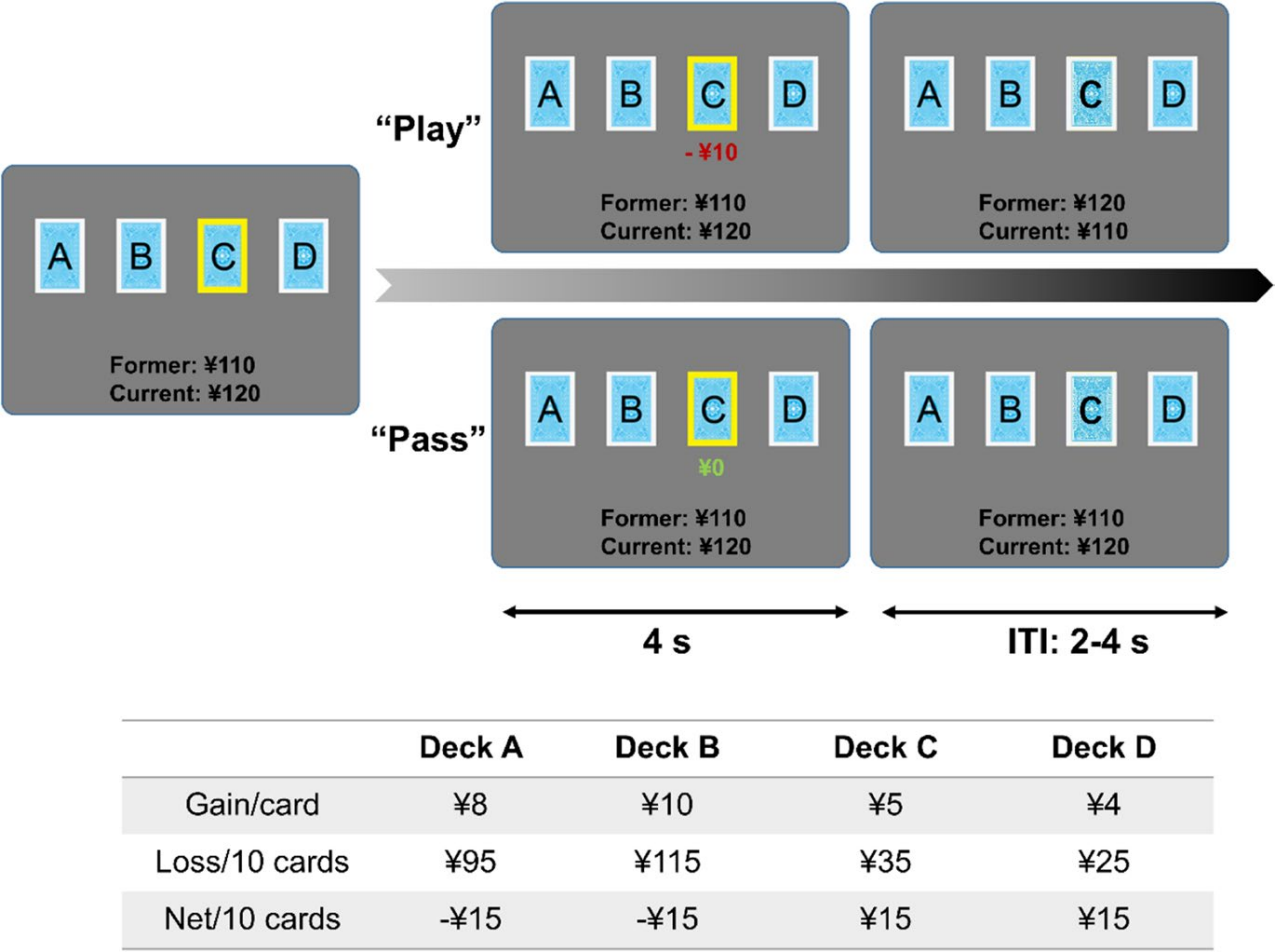
Experimental paradigm of the Iowa gambling task. Each trial began with a yellow frame displayed on one of the four decks randomly. Participants were required to decide whether play or pass the highlighted card as soon as possible within 4 s. Then a feedback with gain/loss was displayed, and total money in the bank was updated. The jittered intertrial interval was between 2 and 4 s. Decks A and B were defined as bad decks with − ¥15 earnings/10 cards, and decks C and D were defined as good decks with ¥15 earnings/10 cards

During the IGT-related fMRI session, a modified IGT was displayed on the screen based on Cauffman and colleague’s version [39]. Participants were required to choose “play”or “pass” the card highlighted with a yellow frame, and were informed that with each selected card, they would win or lose money (a net gain/loss) based on different probability (Fig. 1). Unlike the standard IGT, in which gains per trial are the same for good and bad decks (i.e., $100 for deck A/B, and $50 for deck C/D), the current version uses different gain per trial and corresponding losses for each deck. Out of every ten cards, deck A gave 50% chance of losing ¥95, deck B gave 20% chance of losing ¥115, deck C gave 50% chance of losing ¥35, and deck D gave 20% chance of losing ¥25. Therefore, choosing from the low-risk decks (decks C and D) would give participants a cumulative gain, while choosing from the high-risk decks (decks A and B) would give a loss. With a banked amount of ¥100 at the beginning of the IGT, participants were required to earn monetary reward as much as possible, which means they need to learn the covert rule and optimize their decisions. In each trial, one of the four decks was randomly highlighted with a yellow frame, and participants were required to make a decision (play or pass) as soon as possible. Participants need to make a response within 4 s, then a feedback was displayed showing the corresponding gain or loss in the current trial. If participants chose to play, a feedback with monetary outcome (e.g., + ¥5 or − ¥10) was displayed on the current card. If participants chose to pass, a “¥0” was displayed on the current card. The total number of trials was between 80 and 85, with at least 20 trials for each deck.

After completing the entire task, participant would be paid cash (¥50–¥200) according to the performance. In the behavioral data analysis, the advantageous choice was defined by calculating the proportion of good deck (decks C and D) selections in all the good deck trials, and disadvantageous choice was defined likewise. Then good versus bad selections were used to represent individual’s ability of distinguishing high-risk versus low-risk decks. To examine trajectory of behaviors over time, the entire task was divided into four blocks in the time domain for advantageous and disadvantageous choices, respectively.

### Imaging data acquisition

Neuroimaging data were acquired on a 3.0 T MRI system (Discovery MR750 System, GE Healthcare) with an eight-channel phased-array head coil. The T1-weighted structural images were acquired by using a three-dimensional brain volume imaging sequence that covered the whole brain (repetition time (TR) = 6.7 ms, echo time (TE) = 2.9 ms, flip angle = 7°, matrix = 256 × 256, slice thickness = 1 mm, 196 slices). Then the rs-fMRI data were acquired using gradient-echo echo-planar imaging sequence with the following parameters: TR = 2000 ms, TE = 25 ms, flip angle = 90°, matrix = 64 × 64, and voxel size = 3.4 × 3.4 × 3.2 mm^3^, and 48 axial slices. For each participant, the resting-state fMRI (rs-fMRI) scan duration was 5 min with 150 volumes. During the resting-state session, the participants were required to open their eyes and relax without falling asleep. After a rs-fMRI scanning, a task-related fMRI was delivered using gradient-echo echo-planar imaging sequence with the following parameters: TR = 2000 ms, TE = 25 ms, flip angle = 90°, matrix = 64 × 64, and voxel size = 3.4 × 3.4 × 3.2 mm^3^, and 48 axial slices. During the task session, the IGT task was projected on the screen, and participants made responses by pressing left-handed button for play and right-handed button for pass. The entire task lasted 10 min with 300 volumes.

### Imaging data preprocessing

The functional images were preprocessed in the DPABI_v4.3 (https://rfmri.org/dpabi) [40] based on the SPM8 (http://www.fil.ion.ucl.ac.uk/spm/). For both resting and task data, the first 5 volumes were excluded to obtain steady-state tissue magnetization. The remaining volumes were corrected for slice timing and head motion, co-registered to their own structural images, and normalized to the Montreal Neurological Institute (MNI) standard space. Then the functional images were resampled to 3 × 3 × 3 mm, and smoothed with a Gaussian kernel (FWHM = 6 mm). After preprocessing, two young adults and 6 older adults were removed from the formal analysis due to head motion greater than 2.5 mm or 2.5° during the resting-state or task-related scanning. In addition, given the commonality of head motion in older adults and its confounding effect on resting-state functional connectivity [41, 42]. Framewise displacement, defined as the sum of the absolute values of the six realignment parameters’ derivatives, was calculated for each participant, and controlled as a covariate of non-interest in the following group analyses.

### Gray matter atrophy

Previous studies have provided converging evidence that age plays a major role in brain atrophy as measured by gray matter (GM) volume [43, 44]. The structural images were preprocessed to generate a whole-brain GM map using Voxel-based morphometry (VBM). For each participant, the T1-weighted image was segmented into GM, white matter, and cerebrospinal fluid. Then, a GM template was generated through an iterative nonlinear registration using DARTEL, a toolbox with a fast diffeomorphic registration algorithm [45]. For each participant, an averaged GM volume of the entire brain was computed to control age-induced brain atrophy in the following analyses.

### Resting-state functional connectivity of the fronto-amygdala network

Before functional connectivity analysis, the linear trend was removed, and a bandpass filter (0.01–0.1 Hz) was applied to reduce non-biological signals for resting-state imaging data. Then nuisance covariates were regressed out at the individual level, including 24 head motion parameters, white matter signal, and cerebrospinal fluid signal. The regions of interest (ROIs) including the amygdala, MFC and MOFC were defined based on the AAL atlas [46]. To generate the fronto-amygdala functional network, the BOLD time series within the six regions of interest (ROI) were extracted to correlate with each other and Fisher’s z transformed for each participant. After that, the twelve subnetworks between the amygdala, MFC and MOFC were combined into three subnetworks (i.e., the MOFC-amygdala, MFC-amygdala and MOFC-MFC networks) to simplify the subsequent analysis, regardless of hemisphere. Averaged connectivity strength with each subnetwork was extracted to subsequent analyses. The brain maps were visualized using the BrainNet Viewer Software (http://www.nitrc.org/projects/bnv/).

### Task-related imaging data analysis

A general linear model was applied to examine the BOLD signals linked to six event-related regressors, including the decks A, B, C, D, as well as played and passed decks. Specifically, the regressors were generated by convolving the onset times of each type of events with a canonical hemodynamic response function (HRF). The model also included non-interested regressors as covariates of non-interest in the design matrix, including six head motion parameters, CSF and white matter signals. The trials with no response were excluded, and a high-pass filter with a cut-off period of 128 s was used to remove slow drift. For each participant, contrast images were generated to examine the neural activations in response to good versus bad decks: (deck C + deck D) – (deck A + deck B). Given the complexity of the task, it would be difficult to determine task-related brain activation regardless of the baseline. Therefore, the current analyses focused on interpreting the group differences of good versus bad decks to examine reward/risk estimation. In a ROI-based analysis, six brain regions, including bilateral MFC, MOFC and amygdala, were examined to show the task-evoked BOLD signals in contexts of good, bad, and good versus bad conditions. Averaged BOLD signals within each ROI were extracted to examine the relationships between brain activation and IGT performance. To further examine the subregions within the fronto-amygdala network, a voxel-based analysis was applied within the MFC-MOFC-amygdala mask to examine brain activations in response to good versus bad decks using false discovery rate (FDR) correction (corrected *p* < 0.05 with cluster size > 20 voxels).

### Other statistical analyses

Other statistical analyses were conducted in the Matlab 2014b and SPSS V22. The analysis of covariance (ANCOVA) was used to examine the group differences between young and older adults with education and MMSE as covariates. In the older group, a partial correlation was applied to examine the relationships between IGT performance and functional connectivity/BOLD activity, controlled for age, years of education, GM atrophy, and head motion. A moderation analysis was used to examine the effect of cognitive decline (MMSE/MoCA) in the relationship between age and IGT performance (good versus bad selections), the relationship between functional connectivity/brain activation and IGT performance, as well as the relationship between resting-state connectivity and task-related brain activation, using the PROCESS macro (number of bootstrap samples = 5000) in the SPSS [47]. A generalized linear model (GLM) was used to examine the interaction effect of task-related brain activation and group on IGT performance. In addition, several confounding factors were controlled as covariates in the moderation and GLM analyses, including age, years of education, GM atrophy, and head motion. The FDR correction was used for controlling Type I error in multiple brain region comparisons.

## Results

### Demographic and behavioral data

**Fig. 2:**
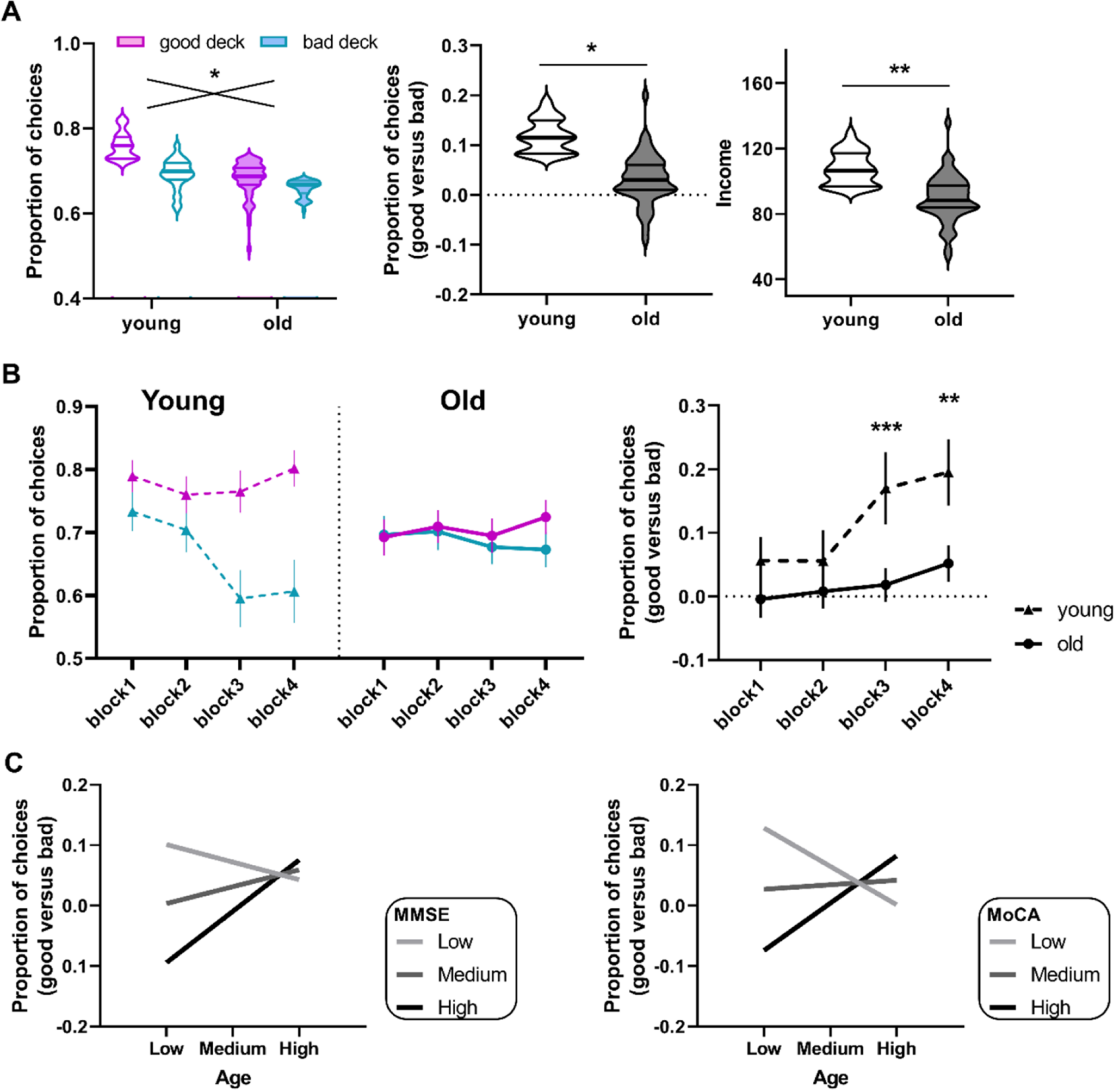
Task performance in the Iowa gambling task. **A** The ANOVA showed a significant interaction between age group and deck (good versus bad) on proportion of choices. Compared with young adults, older adults had difficulties in differentiating good decks from bad decks, leading to significantly lower income in the IGT.** B** During the task, risk-taking behaviors were significantly improved over time in young adults (selecting more good decks and less bad decks), but not in older adults. **C** Moderation analysis showed that age-effect on risk-taking behaviors was depending on the severity of cognitive impairment (MMSE/MoCA), showing a significantly positive correlation between age and task performance (good versus bad selections) in cognitively normal older adults

Before the formal analyses, three older participants were removed due to unreliable IGT performances (i.e., they played or passed all decks). Eventually, thirty-six young and 51 older adults were included in the following analyses. Compared with young adults, older adults have significantly lower MMSE/MoCA scores, and worse IGT performances (Table 1). The ANCOVA showed significant interaction between group and deck (F(1, 83) = 6.19, *p* = 0.015) on deck selections (Fig. 2A). Post-hoc tests showed that significant differences between good versus bad selections in young adults (*t* = 2.86, *p* = 0.007) but not in older adults (*t* = 1.32, *p* = 0.19). Young adults showed significantly better decision (selecting more good decks over bad decks) (F (1, 83) = 6.22, *p* = 0.015) and higher income (F(1, 83) = 6.8.02, *p* = 0.006) than older adults. In the block-based analysis (Fig. 2B), the trajectories of IGT performance exhibited the differences in reinforcement learning between young and older adults. A significant interaction effect between deck and block was observed in the young group (F(3, 105) = 4.33, *p* = 0.011) but not in the older group (F(3, 150) = 2.19, *p* = 0.11), showing less selections of bad deck in younger adults (Fig. 2B left). The multivariate ANOVA showed that young adults showed significantly better good versus bad selections in the last two blocks (block 1: F(1, 83) = 2.46, *p* = 1.21; block 2: F(1, 83) = 2.59, *p* = 0.004; block 3: F(1, 83) = 9.67, *p* = 0.003; block 4: F(1, 83) = 7.87, *p* = 0.006) compared with older adults (Bonferroni correction, Fig. 2B right). The above group comparisons of task behavior were adjusted for years of education and MMSE. In addition, older individuals showed marginal significance between good and bad selections in the last block (block 4: *t* = 1.81, *p* = 0.077), and slightly improved their decisions over time (block 4 versus block 1: *t* = 1.96, *p* = 0.055). The results indicated that young adults could successfully differentiate between good and bad decks to optimize their decisions, which was considerably impaired in older adults.

**Table 1:** Demographic, neuropsychological data, and performance in the IGT

	Young (*n* = 36)	Old (*n* = 51)	*p* value
Age	23.31 ± 2.11	64.29 ± 5.44	** 0.000**
Male/Female	22/14	26/25	0.353
EDU (years)	12.86 ± 2.07	11.91 ± 2.37	0.056
MMSE	29.33 ± 0.89	27.78 ± 2.03	** 0.000**
MOCA	27.50 ± 1.44	22.51 ± 3.38	** 0.000**
Iowa gambling task			
Good selection	0.78 ± 0.14	0.71 ± 0.20	0.066
Bad selection	0.66 ± 0.20	0.68 ± 0.19	0.265
Good vs. Bad selection	0.12 ± 0.25	0.03 ± 0.17	**0.015**
Income	107.76 ± 38.05	89.75 ± 46.88	**0.006**

Data are presented as means ± standard deviations. The independent *t*-test and *χ*^2^-test were used to examine group differences in age, gender, years of education, MMSE, and MoCA. The ANCOVA were used to examine the differences between young and older adults in the IGT, controlled for years of education and MMSE

*MMSE* Mini-mental State Examination, *MOCA* Montreal Cognitive Assessment, *IGT* Iowa gambling task

*p* values in bold are presented with *p* < 0.000

The moderation analysis was used to examine the effect of cognitive integrity between age and risk-taking behaviors in older adults, controlled for age and years of education (Fig. 2C**)**. Specifically, MMSE significantly moderated the age-effect on good versus bad selections (*b* = 0.01, se = 0.002, *t* = 2.34, *p* = 0.024, 95% CI [0.004, 0.03]), showing a significant positive relationship between age and behaviors in older adults with high MMSE scores (*b* = 0.02, se = 0.01, *t* = 2.60, *p* = 0.013, 95% CI [0.001, 0.01]), and no relationship in older adults with low MMSE scores (*b* =  − 0.05, se = 0.01, *t* =  − 0.84, *p* = 0.41, 95% CI [− 0.02, 0.01]). Likewise, MoCA scores moderated the relationship between age and good versus bad choice (*b* = 0.004, se = 0.001, *t* = 3.04, *p* = 0.004, 95% CI [0.001, 0.01]), showing a significant positive relationship between age and behaviors in high MoCA scores (*b* = 0.01, se = 0.005, *t* = 2.80, *p* = 0.004, 95% CI [0.004, 0.03]), and no relationship in low MoCA scores (*b* =  − 0.01, se = 0.007, *t* =  − 1.63, *p* = 0.11, 95% CI [− 0.03, 0.003]).

### Resting-state fronto-amygdala connectivity analysis

**Fig. 3:**
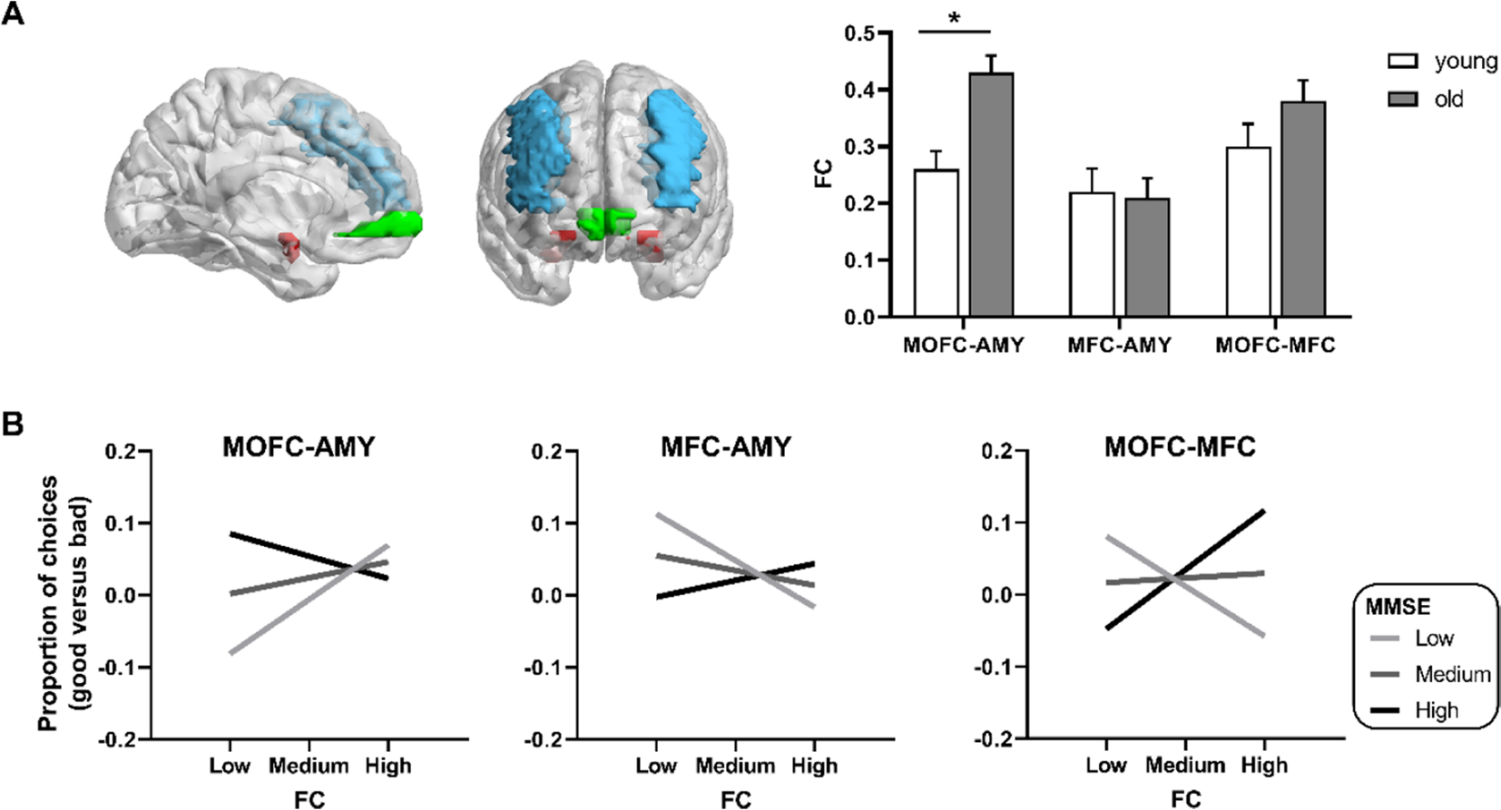
The resting-state fronto-amygdala connectivity associated with IGT performance. **A** In resting-state functional connectivity analysis, the ROIs (i.e., left and right amygdala, MFC, and MOFC) were derived from the AAL atlas. In the multivariate ANOVA, older adults showed significantly increased connectivity strength within the MOFC-amygdala network, but not within the MFC-amygdala and MOFC-MFC networks, compared with young adults. **B** In the older group, a moderation analysis was conducted to examine whether the relationship between functional connectivity and IGT performance (good versus bad selections) was influenced by age-related cognitive decline, controlled for age, education, GM atrophy and head motion. MMSE scores moderated the relationship between MOFC-amygdala connectivity and good versus bad selections, showing a positive correlation in low-MMSE scores. In contrast, MMSE scores moderated the relationship between MFC-amygdala connectivity and task performance, showing a negative correlation in low MMSE scores. In addition, MMSE scores moderated the relationship between MOFC-MFC connectivity and task performance, showing a negative correlation in low MMSE scores and positive correlation in high MMSE scores. Abbreviations: AMY, amygdala; GM, gray matter; MFC, middle frontal cortex; MOFC, medial orbitofrontal cortex

In the ROI-based analysis, the six ROIs (i.e., the left and right amygdala, MOFC, and MFC) in bilateral hemispheres were derived from the AAL (Fig. 3A left). By calculating the functional connectivity between the ROIs, twelve subnetworks between each pair of ROIs were generated, excluding the left amygdala-right amygdala, left MOFC-right MOFC as well as left MFC-right MFC networks. To simplify the analysis, three networks (i.e., MOFC-amygdala, MFC-amygdala, and MOFC-MFC networks) were defined by averaging the connectivity strength across the hemispheres. In Fig. 3A right, the multivariate ANOVA showed that older adults showed a significantly decreased connectivity strength in the MOFC-amygdala network (F(1, 81) = 4.04, *p* = 0.048), but not in the MFC-amygdala (F(1, 83) = 0.24, *p* = 0.63) or MOFC-MFC networks (F(1, 83) = 1.41, *p* = 0.24), controlled for education, MMSE, GM atrophy, and head motion (Bonferroni correction).

In older adults, we assessed the moderating effects of cognitive integrity defined by MMSE scores on the relationship between functional connectivity and IGT performance (good versus bad selections), controlled for education, age, GM atrophy, and head motion. For the MOFC-amygdala network (Fig. 3B left), MMSE scores significantly moderated the relationships between functional connectivity and IGT performance (*b* =  − 0.12, se = 0.03, *t* =  − 3.57, *p* = 0.001, 95% CI [− 0.19, − 0.05]). In individuals with low MMSE scores, functional connectivity was positively associated with IGT performance (*b* = 0.39, se = 0.11, *t* = 3.57, *p* < 0.001, 95% CI [0.17, 0.61]). In individuals with high MMSE scores, no correlation was observed (*b* =  − 0.11, se = 0.13, *t* =  − 0.82, *p* = 0.41, 95% CI [− 0.37, 0.16]).

For the MFC-amygdala network (Fig. 3B middle), MMSE scores significantly moderated the relationships between functional connectivity and IGT performance (*b* = 0.09, se = 0.04, *t* = 2.31, *p* = 0.026, 95% CI [0.01, 0.17]). In low MMSE scores, functional connectivity was negatively associated with IGT performance (*b* =  − 0.24, se = 0.12, *t* =  − 2.03, *p* = 0.048, 95% CI [− 0.47, − 0.002]). In high MMSE scores, no correlation was observed (*b* = 0.12, se = 0.13, *t* = 0.95, *p* = 0.35, 95% CI [− 0.14, 0.39]).

In addition, for the MOFC-MFC network (Fig. 3B right), MMSE scores significantly moderated the relationships between functional connectivity and IGT performance (*b* = 0.14, se = 0.03, *t* = 4.48, *p* < 0.001, 95% CI [0.08, 0.21]). Further analysis showed that correlation between functional connectivity and IGT performance was negative in low MMSE scores (*b* =  − 0.25, se = 0.09, *t* =  − 2.67, *p* = 0.01, 95% CI [−0.44, − 0.06]), and was positive in high MMSE scores (*b* = 0.34, se = 0.11, *t* = 3.14, *p* = 0.003, 95% CI [0.12, 0.56]).

**Fig. 4:**
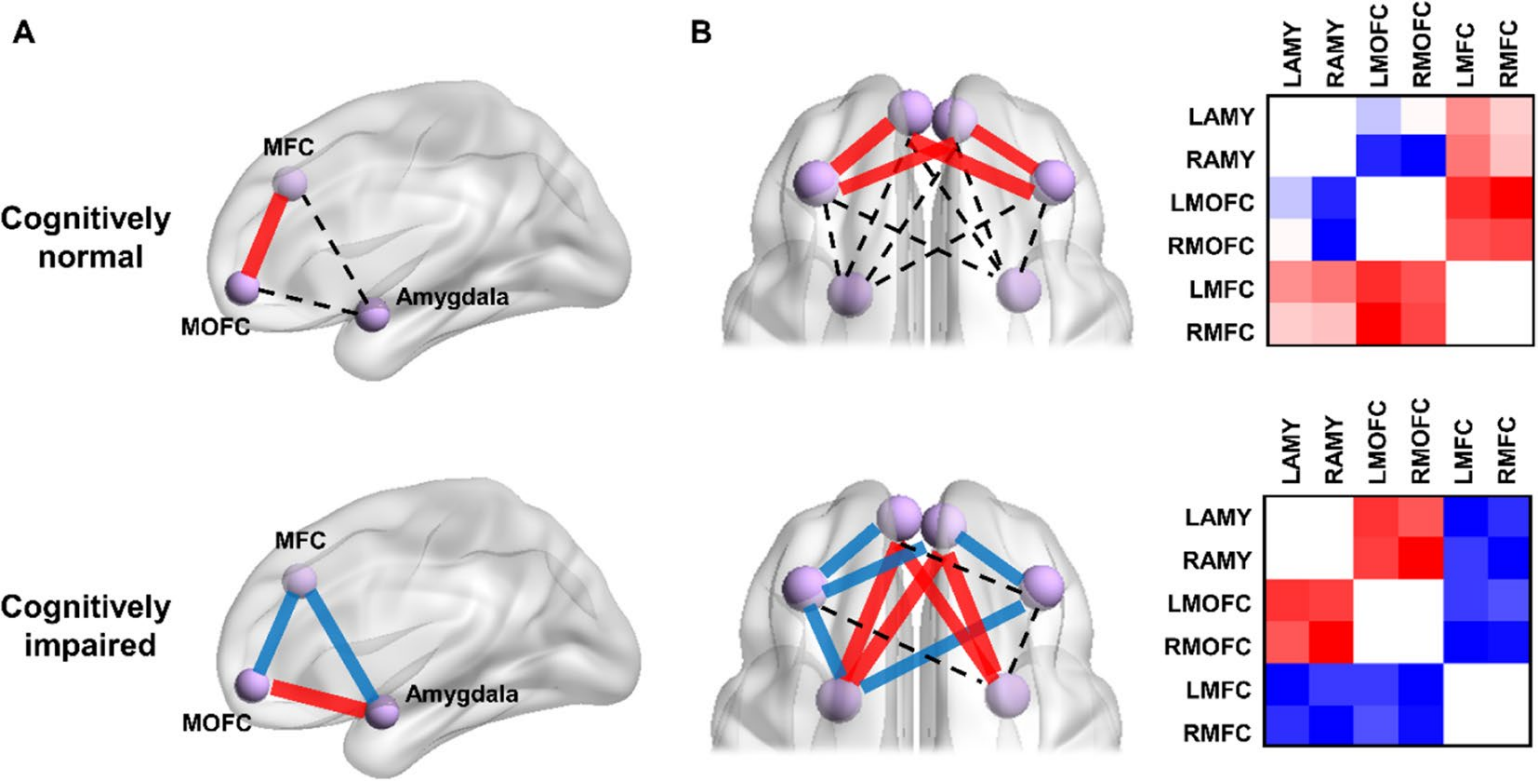
The neural framework for representing individual difference of risk-taking behaviors in aging. **A** The moderation results were visualized by showing the relationships between fronto-amygdala connectivity and IGT performance (good versus bad selections) in cognitively normal and impaired older adults, separately. The patterns of positive (red lines) and negative correlations (blue lines) were remarkably changed in cognitively impaired individuals. And no correlation is shown in dashed line. **B** Using 12 subnetworks, the moderation effect of MMSE was opposite in cognitively normal and impaired individuals. The correlation patterns between functional connectivity and IGT performance were shown in coefficient matrices in the left column

To summarize, compared with cognitively normal older adults (high MMSE scores), risk-taking behavior was more closely associated with the fronto-amygdala connectivity in cognitively impaired individuals (low MMSE scores), showing an opposite pattern of correlation in the dorsal and ventral pathways. Based on the aforementioned moderation models, a framework is built for describing the neural correlates underlying individual differences of risk-taking behavior in aging, that the dorsal and ventral fronto-amygdala pathways were associated with IGT performance differently in the context of cognitive decline (Fig. 4A). Furthermore, additional analyses were performed to examine the patterns of moderation effects with 12 subnetworks, showing the same correlation patterns in the context of cognitive decline (Fig. 4B, Table S1, and Figure S1). Consistent with the findings in MMSE-based analyses, the relationships between the fronto-amygdala network and risk-taking behaviors were moderated via MoCA scores as well (Figure S2).

### Task-related brain activation

**Fig. 5:**
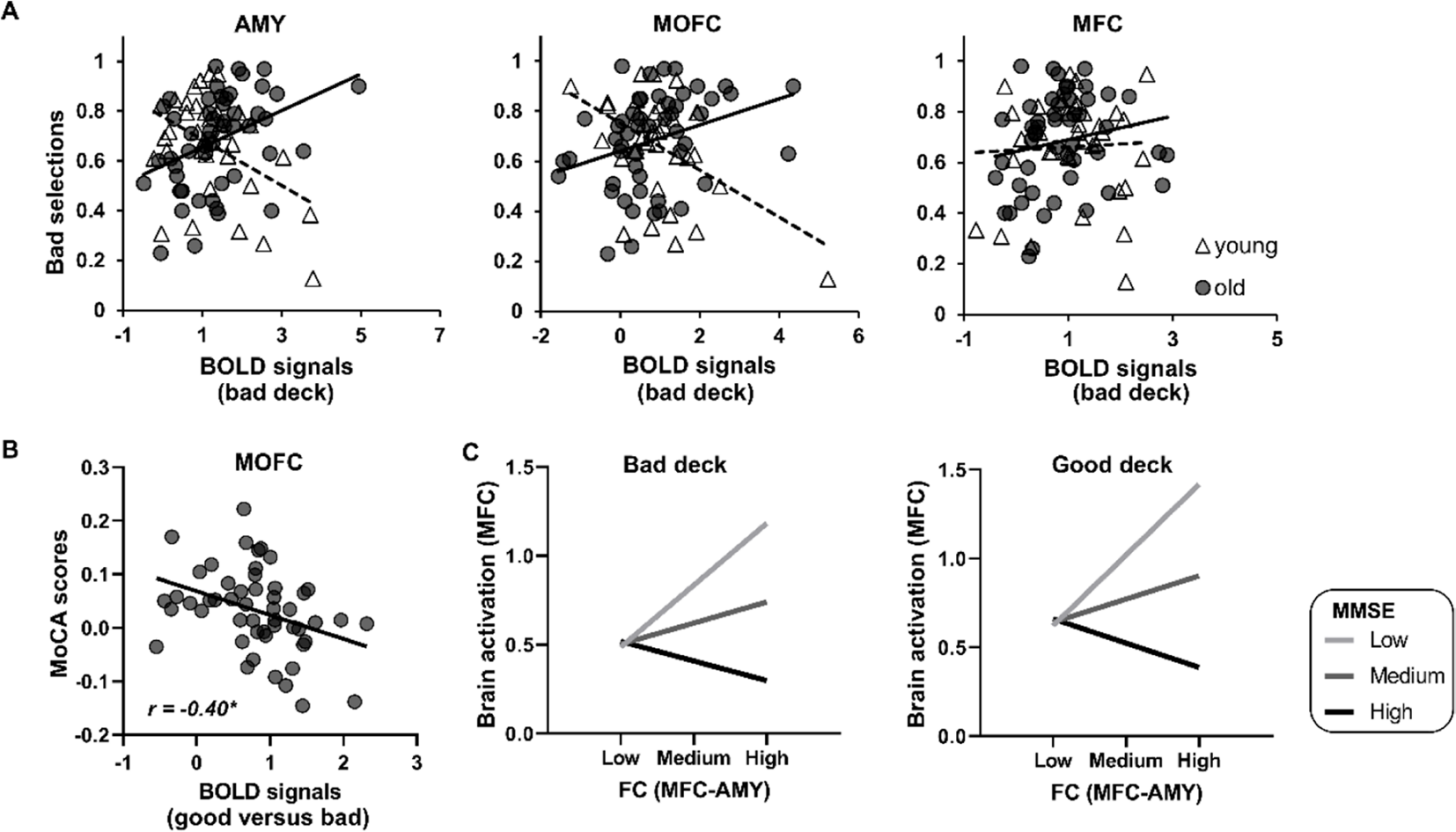
ROI-based analysis in the task-related fMRI. **A** Compared with young adults, older adults showed significantly changed correlations between bad selections and brain activation in the amygdala and MOFC. **B** In the moderation analyses, resting-state MFC-amygdala connectivity significantly correlated to task-related MFC activation in cognitively impaired older adults, but not in cognitively normal older adults. *p < 0.05

For each participant, a contrast map of (deck C + deck D) – (deck A + deck B) was generated to measure the differences of brain activation between good and bad decks. In ROI-based analysis, averaged BOLD activity within the three ROIs (i.e., the amygdala, MOFC, and MFC) were extracted to examine the relationships with cognitive decline and IGT performance. In the GLMs, a significant positive correlation between selections of bad decks and amygdala activity was observed in older adults (b(se) = 0.17 (0.04), Wald χ^2^(1) = 18.87, *p* < 0.001), compared with young adults (Fig. 5A). Likewise, a similar interaction effect between group and brain activation on bad selections was observed in the MOFC (b(se) = 0.15 (0.04), Wald χ^2^(1) = 18.87, *p* < 0.001), but not in the MFC (b(se) = 0.06 (0.05), Wald χ^2^(1) = 1.04, *p* = 0.31).

In the older group, further analyses were performed to examine whether task-related brain activation was correlated to task behavior and resting-state functional connectivity. In Fig. 5B, the partial correlation showed MOFC activity was negatively correlated with MoCA scores (*r* =  − 0.40, *p* = 0.015), but not correlated with MMSE scores (*r* =  − 0.21, *p* = 0.22) (FDR correction). In the condition of bad deck, MMSE scores significantly moderated the relationship between MFC-amygdala connectivity and MFC activation (*b* =  − 0.48, se = 0.17, *t* =  − 2.90, *p* = 0.006, 95% CI [− 0.82, − 0.15]), showing a positive correlation between BOLD activity and connectivity in lower MMSE scores (*b* = 1.36, se = 0.50, *t* = 2.71, *p* = 0.01, 95% CI [0.34, 2.38]) and no correlation in higher MMSE scores (*b* =  − 0.60, se = 0.57, *t* =  − 1.05, *p* = 0.30, 95% CI [− 1.75, 0.55]). In the condition of good deck, MMSE scores significantly moderated the relationship between MFC-amygdala connectivity and MFC activation (*b* =  − 0.54, se = 0.16, *t* =  − 3.31, *p* = 0.004, 95% CI [− 0.87, − 0.21]), showing a positive correlation between BOLD activity and connectivity in lower MMSE scores (*b* = 1.65, se = 0.50, *t* = 3.32, *p* = 0.002, 95% CI [0.65, 2.65]) and no correlation in higher MoCA scores (*b* =  − 0.56, se = 0.56, *t* =  − 1.01, *p* = 0.32, 95% CI [− 1.69, 0.56]) (Fig. 5C).

**Fig. 6:**
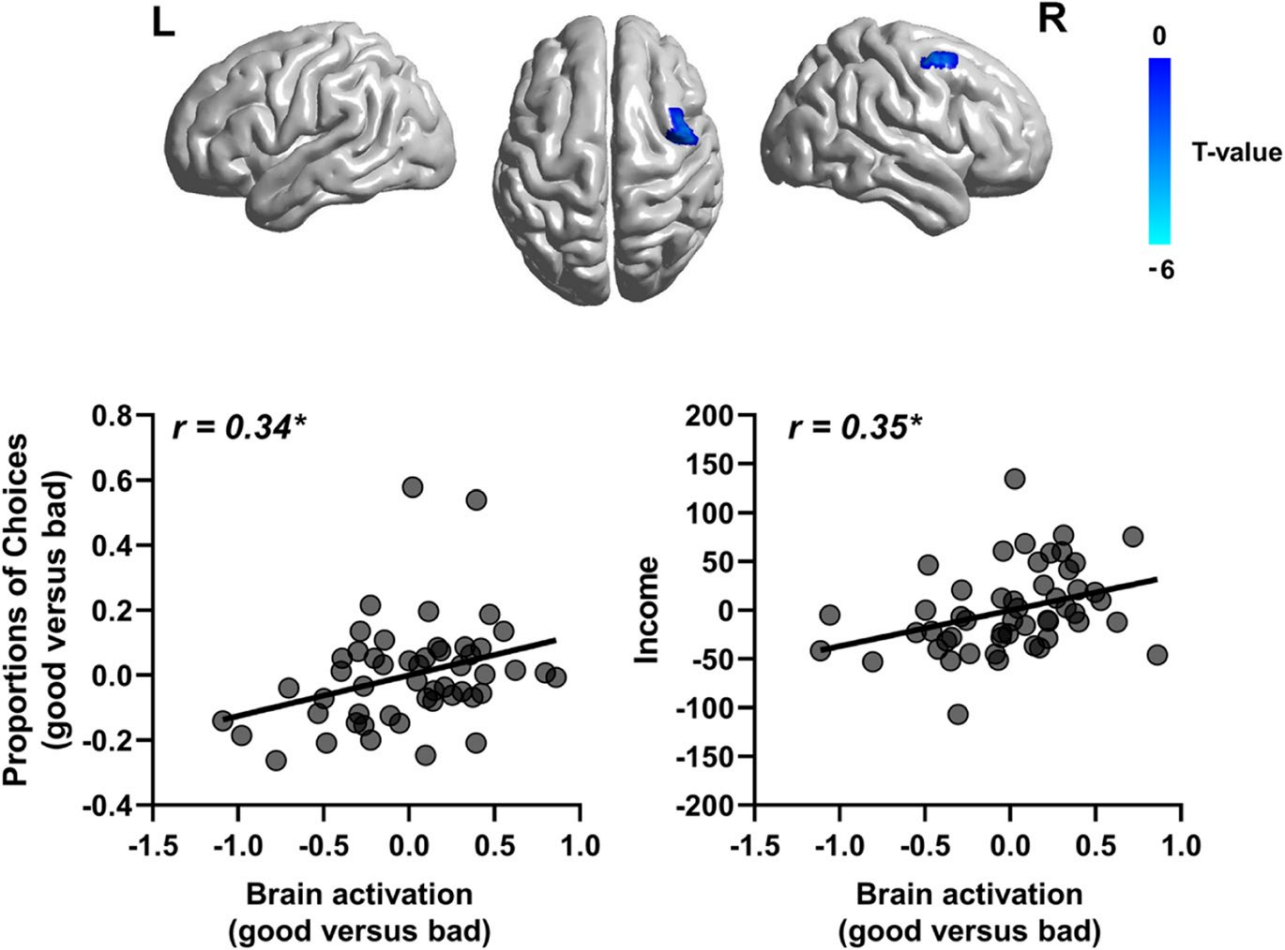
Voxel-based analysis in the task-related fMRI. Within the fronto-amygdala network, a subregion of the MFC showed positive correlations between brain activation and IGT performance (both good versus bad selections and income). *p < 0.05

In the voxel-based analysis, a right MFC subregion (T-value =  − 5.61, Brodmann’s area 6, MNI: 48, 0, 54, cluster = 32 voxels) within the MFC-MOFC-amygdala mask showed significantly different BOLD activation between good and bad decks (Fig. 6). Partial correlation analyses showed that activation of the MFC subregion was positively correlated with good versus bad selections (*r* = 0.34, *p* = 0.021), as well as total income (*r* = 0.35, *p* = 0.015), controlled for education, head motion, and GM atrophy.

## Discussion

To the best of our knowledge, this is the first study to investigate the relationship between risky decision-making and the fronto-amygdala network in older adults with different severity of cognitive decline. Compared with young adults, older adults exhibited remarkable changes in risk-taking behavior and corresponding fronto-amygdala circuitry. Age-related cognitive decline did not impact risky decision-making directly, however, it considerably moderated the relationship between risk-taking behavior and corresponding brain activity in older adults. The relationships between intrinsic fronto-amygdala network and risk-taking behavior were significantly different in cognitively normal and impaired older adults, showing that the dorsal and ventral PFC interacting with the amygdala made dissociable contributions to reward-based decision strategy. Task-related fMRI analysis confirmed the critical role of the fronto-amygdala network in decision making under uncertainty depending on the severity of cognitive decline. Our findings imply that the amygdala was functionally coupled to specific prefrontal areas involved in two crucial neural pathways in modulating risky decision-making, underlying individual differences in strategy formation and risk-taking behavior in older adults.

The behavioral data showed that young adults could successfully differentiate good decks from bad decks to optimize their decisions over time, which confirmed the reliability of the customized IGT. Compared with the young group, the older group exhibited remarkably changed risk preferences and reinforcement learning especially in the bad decks, showing less sensitivity to risk in processing reward/loss under uncertainty. In line with our findings, older adults relative to young adults were likely to make more risk-seeking mistakes in a dynamic financial investment task [9]. However, the learning curve of good-bad choice was gradually enhanced over time, suggesting that older adults were still capable of learning covert rules and improving their strategies [12]. Furthermore, moderation analyses showed a positive age-effect on good versus bad choice in cognitively normal older adults, but not in cognitively impaired individuals. Consistently, Rutledge et al. have reported that risk-seeking behaviors were observed to be decreased in trials with potential gains, but increased in mixed trials with both gains and losses over the lifespan [4]. Some researchers argued that accumulated knowledge and less negative emotion could benefit financial decision making in older adults [48, 49]. These results suggest that risk-taking behavior may not be monotonically changed in natural aging, and individual differences in risk-taking behaviors in aging might be influenced by cognitive ability.

In the resting-state fMRI analysis, the connectivity strength of the MOFC-amygdala network was significantly increased in older adults, compared with young adults. More importantly, the MFC-amygdala and MOFC-amygdala connections were observed to be associated with risk-taking behavior differently in the context of cognitive decline. In specific, older adults with cognitive decline showed good versus bad selections associated with functional connectivity positively in the MOFC-amygdala network and negatively in the MFC-amygdala network, respectively. Here the opposite directions of the correlations suggest dissociable contributions of the dorsal and ventral frontal regions interacting with the amygdala in regulating risky decision-making in aging. Previous studies showed that the stronger ventral medial PFC-amygdala connectivity was associated with enhanced risk-tolerance [3], and led to better performance (more reward) under risk [50]. Orsini et al. have reported that the OFC lesions increased risk-aversive behaviors and amygdala lesion increased risk-seeking behaviors [30]. Zeeb et al. also showed that disconnecting the OFC and amygdala prevented learning appropriate assessment of reward value in a rodent analogue of the IGT [26]. Therefore, enhanced intrinsic MOFC-amygdala connectivity in low cognitive scores, which was correlated with better task performance, might compensate for cognitive decline to maintain optimal decision strategy. In contrast, the changed correlations between the MFC-amygdala and risk-taking behavior from high to low cognitive functioning suggest a miscommunication in the dorsal fronto-amygdala network. It has been well documented that the MFC subregions such as DLPFC is critical for impulsive control and decision making, facilitating to pursue long-term over immediate rewards [31, 51]. In addition to the fronto-amygdala connectivity, the MFC-MOFC connectivity was associated with risk-taking behaviors in cognitively normal and impaired older adults differently. Previous studies have shown that the decoupling of MFC/DLPFC with medial PFC is consistent with the impaired executive functioning in bipolar and schizophrenia disorders [52], while increased functional coupling between these two regions could benefit behavioral control in children and memory-dependent decision in older adults [53, 54]. In conclusion, we speculate that the reversed correlations between MFC-amygdala/MFC-MOFC connections and risk-taking behavior in cognitively decline might be due to impaired fontal functioning in the aging brain, while MOFC-amygdala connectivity might compensate these dysfunctions to maintain normal decision strategy despite of cognitive decline. In line with our findings, previous studies have reported that impairment and compensation coexist in brain activity/connectivity in older adults with cognitive impairments [55, 56]. Therefore, older adults might still learn covert rules in uncertainty task by reallocating the weights of the fronto-amygdala network in response to cognitive decline.

In addition to resting-state fMRI analysis, task-related fMRI data was used to further examine the fronto-amygdala network during the IGT. In the condition of bad deck, the correlations between bad selections and brain activations (within the amygdala and MOFC but not the MFC) were significantly changed in older adults relative to young adults, confirming our hypothesis that dorsal and ventral neural path may contribute differently in aging. Interestingly, the moderation analyses exhibited that intrinsic MFC-amygdala connectivity was significantly correlated with task-related brain activation in cognitively impaired older adults, but not in cognitively normal individuals. We speculated that the abnormally enhanced correlation might represent less complex and more predictable brain signals in age-related cognitive impairment [57], which should be further investigated in the future. In additions, the positive correlation between bad selections and amygdala activation suggests more risk-seeking behaviors with greater amygdala activation in aging, confirming the group difference of task performance in our behavioral results. In line with our findings, Jung et al. reported that amygdala is representative of “cost” in reward-based task, and greater amygdala activity/connectivity is associated with higher risk tolerance [3]. For good versus bad decks, the negative correlations between MoCA scores and MOFC activation suggests that MOFC in response to reward/risk estimation (differentiating good versus bad decks) was influenced in cognitive decline. Animal and human studies have consistently shown that MOFC functioning is critical for forming optimal strategies in reward-guided decision making [58, 59]. In voxel-wise analysis, a MFC subregion was activated in the condition of good versus bad decks, showing significant positive correlations with risk-taking strategy as well as task income, in line with the study showing MFC activation associated with task-related risk taking and reinforcement learning [2]. Here we did not observe significant correlation between task-evoked MOFC activation and risk-taking behavior in both ROI- and voxel-based analysis, in line with the negative findings in the relationship between OFC and advantageous versus disadvantageous choices [60–62]. However, this may be due to the complex neurobiological architecture of the OFC, more efficient analysis tools such as multiple variate pattern analysis need to be considered in future research [20, 63].

Several limitations of the current study should be acknowledged. First, the current study only investigated the interactions between the amygdala, MOFC and MFC in the fronto-amygdala network. Other brain regions/networks should be involved in future research to validate our findings, especially the core regions involved in the reward system such as striatum and insula. Second, the current study included older adults with a large range of MoCA/MMSE scores to examine task behaviors and brain activations in different severity of cognitive decline. Those participants with low cognitive scores might have high risk of MCI. Although it is still unknown whether normal aging and MCI are significantly different in risky decision-making [1, 64, 65], it is worthwhile to further examine the fronto-amygdala circuitry at different stages of MCI (i.e., early and late). Third, other popular analysis methods such as machine learning have been widely used in neuroimaging studies as well, thus a larger study cohort with more participants would help validate our findings in the future. Lastly, the current analyses treated the amygdala as a single homogeneous structure, however, the amygdala is a collection of nuclei known to have different functions and patterns of connectivity [66]. Future research is needed to investigate more functional networks involving amygdala subregions, such as lateral and basal nucleus.

## Conclusion

In summary, the current study investigated the relationship between risk-taking behavior and the fronto-amygdala network in the context of age-related cognitive decline. We found that the dorsal and ventral fronto-amygdala connectivity predicted risk-taking behavior in older adults. The distinct patterns of correlations in the dorsal and ventral neural circuits suggest their dissociable contributions in value-based decision making, which was changed in age-related cognitive decline. We speculated that older adults with cognitive decline might still be capable of forming optimal decision strategy in reward-based decision task by reallocating the fronto-amygdala connections. Collectively, our findings imply that the fronto-amygdala network may be critical for understanding individual differences in risk-taking behaviors in aging.

### Supplementary Information

Below is the link to the electronic supplementary material.

Supplementary file1 (DOCX 206 KB)

## Data Availability

The data used in this study is available from the corresponding author upon request.
