# Supplementary material for: Dorsal and ventral fronto-amygdala networks underlie risky decision-making in age-related cognitive decline
Source: GeroScience. 2023 Sep 12;46(1):447–62. doi: 10.1007/s11357-023-00922-2 (PMC10828304; doi:10.1007/s11357-023-00922-2)

| Table S1. Moderation analysis. | | | | | |
| --- | --- | --- | --- | --- | --- |
|  | **Coeff** | **SE** | **t** | **p** | **95%CI** |
| *Left MOFC-left AMY* |  |  |  |  |  |
| Interaction | -0.10 | 0.03 | -3.21 | **0.003** | [-0.16, 0.04] |
| Low MMSE | 0.35 | 0.10 | 3.59 | **<0.001** | [0.15, 0.55] |
| High MMSE | -0.05 | 0.11 | -0.41 | 0.69 | [-0.29, 0.19] |
| *Right MOFC-left AMY* |  |  |  |  |  |
| Interaction | -0.07 | 0.02 | -3.28 | **0.002** | [-0.11, -0.03] |
| Low MMSE | 0.29 | 0.08 | 3.90 | **<0.001** | [0.14, 0.45] |
| High MMSE | 0.01 | 0.10 | 0.10 | 0.92 | [-0.20, 0.22] |
| *Left MFC-Left AMY* |  |  |  |  |  |
| Interaction | 0.10 | 0.03 | 2.91 | **0.006** | [0.03, 0.17] |
| Low MMSE | -0.23 | 0.11 | -1.98 | 0.054 | [-0.46, 0.00] |
| High MMSE | 0.17 | 0.12 | 1.44 | 0.16 | [-0.07, 0.41] |
| *Right MFC-Left AMY* |  |  |  |  |  |
| Interaction | 0.07 | 0.03 | 2.43 | **0.019** | [0.01, 0.12] |
| Low MMSE | -0.19 | 0.09 | -2.03 | **0.048** | [-0.38, 0.00] |
| High MMSE | 0.08 | 0.12 | 0.68 | 0.50 | [-0.16, 0.32] |
| *Left MOFC-Right AMY* |  |  |  |  |  |
| Interaction | -0.13 | 0.05 | -2.52 | **0.016** | [-0.23, -0.03] |
| Low MMSE | 0.33 | 0.15 | 2.20 | **0.03** | [0.03, 0.63] |
| High MMSE | -0.19 | 0.14 | -1.35 | 0.18 | [-0.47, 0.09] |
| *Right MOFC-Right AMY* |  |  |  |  |  |
| Interaction | -0.16 | 0.05 | -3.56 | **<0.001** | [-0.26, -0.07] |
| Low MMSE | 0.44 | 0.13 | 3.34 | **0.002** | [0.18, 0.71] |
| High MMSE | -0.22 | 0.13 | -1.69 | 0.10 | [-0.49, 0.04] |
| *Left MFC-Right AMY* |  |  |  |  |  |
| Interaction | 0.06 | 0.31 | 1.88 | 0.067 | [-0.004, 0.12] |
| Low MMSE | -0.18 | 0.13 | -1.39 | 0.17 | [-0.44, 0.08] |
| High MMSE | 0.21 | 0.14 | 1.50 | 0.14 | [-0.07, 0.48] |
| *Right MFC-Right AMY* |  |  |  |  |  |
| Interaction | 0.05 | 0.03 | 1.86 | 0.070 | [-0.004, 0.10] |
| Low MMSE | -0.24 | 0.11 | -2.18 | 0.035 | [-0.46, -0.02] |
| High MMSE | 0.09 | 0.13 | 0.73 | 0.47 | [-0.16, 0.35] |
| *Left MOFC-left MFC* |  |  |  |  |  |
| Interaction | 0.13 | 0.03 | 3.75 | **<0.001** | [0.06, 0.20] |
| Low MMSE | -0.18 | 0.19 | -2.00 | 0.052 | [-0.37, 0.00] |
| High MMSE | 0.33 | 0.11 | 3.01 | **0.004** | [0.11, 0.54] |
| *Right MOFC-left MFC* |  |  |  |  |  |
| Interaction | 0.13 | 0.03 | 4.38 | **<0.001** | [0.70, 0.18] |
| Low MMSE | -0.24 | 0.08 | -2.87 | **0.006** | [-0.41, -0.07] |
| High MMSE | 0.27 | 0.09 | 2.82 | **0.007** | [0.08, 0.46] |
| *Left MOFC-Right MFC* |  |  |  |  |  |
| Interaction | 0.14 | 0.03 | 4.13 | **<0.001** | [0.07, 0.21] |
| Low MMSE | -0.16 | 0.09 | -1.87 | 0.068 | [-0.34, 0.01] |
| High MMSE | 0.40 | 0.11 | 3.52 | **0.001** | [0.17, 0.63] |
| *Right MOFC-Right MFC* |  |  |  |  |  |
| Interaction | 0.13 | 0.03 | 4.36 | **<0.001** | [0.07, 0.18] |
| Low MMSE | -0.22 | 0.08 | -2.72 | **0.009** | [-0.38, -0.06] |
| High MMSE | 0.29 | 0.11 | 2.70 | **0.01** | [0.07, 0.50] |

For each of the 12 subnetworks, a moderation analysis was performed to examine the effect of MMSE in the relationship between functional connectivity and IGT performance (good versus bad selections). The coefficients of the relationships between functional connectivity and task behavior were shown in individuals with low MMSE (Mean-1SD) and high MMSE (Mean+1SD) scores, respectively. Abbreviations: MMSE, Mini Mental State Examination; MFC, middle frontal cortex; MOFC, medial orbitofrontal cortex.

**Figure S1.** The moderating effect of MMSE between functional connectivity and IGT performance in older adults. To comprehensively examine the influence of cognitive ability, moderation analysis was performed for 12 subnetworks. **A)** Regardless of hemisphere, MMSE significantly moderated the relationships between MOFC- amygdala connectivity and good-bad choice, showing a significantly positive relationship between connectivity and good-bad choice in low MMSE scores, and no relationship in high MMSE scores. **B)** In contrast, MMSE significantly moderated the relationships between left/right MFC-left amygdala connectivity and good-bad choice, showing negative correlation between connectivity and good-bad choice in low MMSE scores, and no correlation in high MMSE scores. **C)** For the MOFC-MFC networks, MMSE significantly moderated the relationships between functional connectivity and good-bad choice, showing negative or no correlation in low MMSE scores, and significantly positive correlation in high MMSE scores.

**
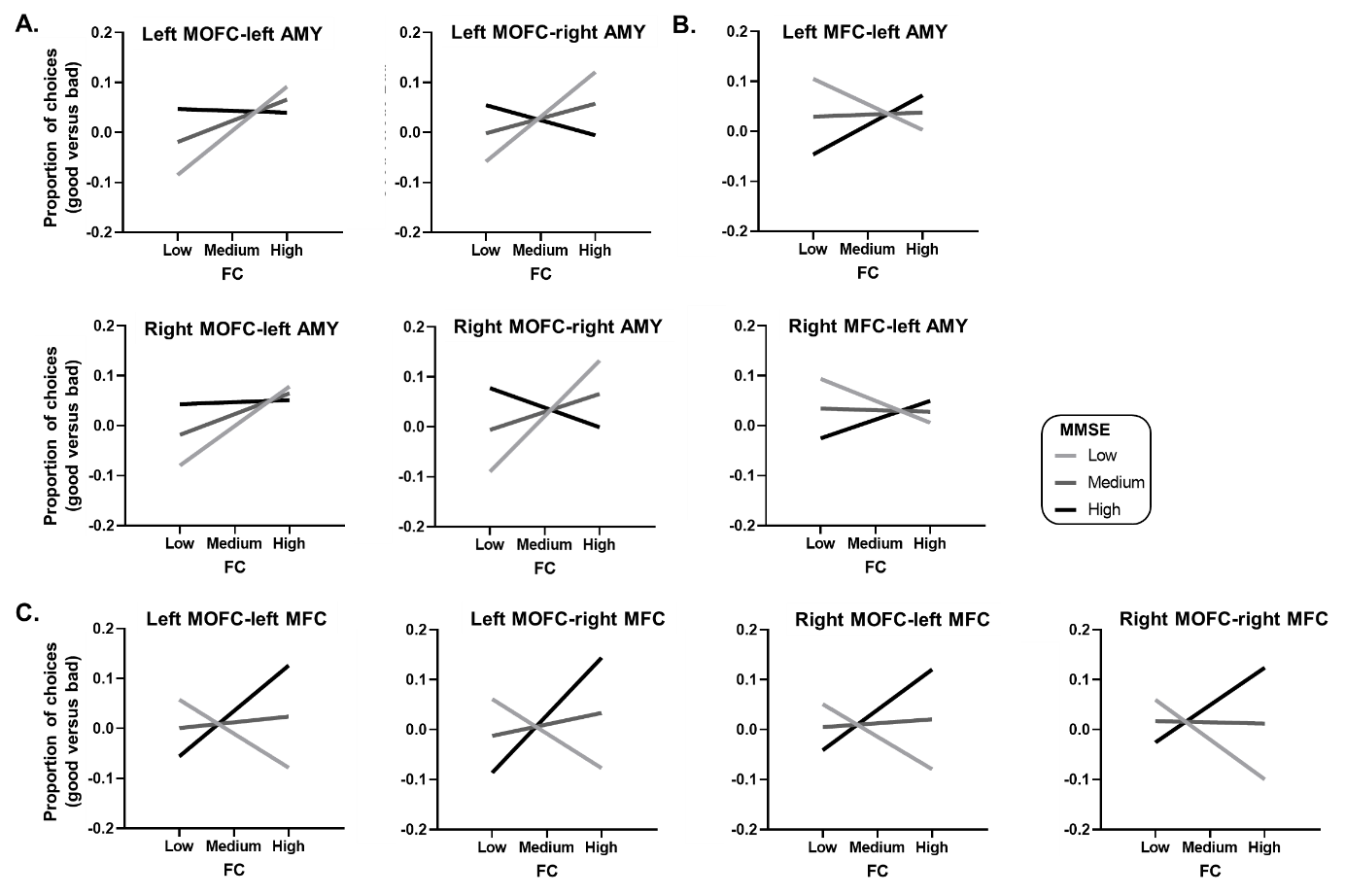
**

**Figure S2.** The moderating effect of MoCA between functional connectivity and IGT performance in older adults. MoCA significantly moderated the relationship between MOFC- amygdala connectivity and good versus bad choice (b = -0.06, se = 0.02, t = -2.72, p = 0.009, 95%CI [-0.11, -0.02]), showing a significant positive correlation between connectivity and behaviors in low MoCA scores (b = 0.39, se = 0.11, t = 3.29, p = 0.002, 95%CI [0.15, 0.63]) and no correlation in high MoCA scores (b = -0.05, se = 0.14, t = -0.32, p = 0.75, 95%CI [-0.33, 0.24]). In contrast, the moderating effect of MoCA were observed between MFC- amygdala connectivity (b = 0.08, se = 0.03, t = 2.86, p = 0.011, 95%CI [0.02, 0.14]), showing a negative correlation in low MoCA scores (b = -0.31, se = 0.13, t = -2.47, p = 0.017, 95%CI [-0.57, -0.06]), and no correlation high MoCA scores (b = 0.26, se = 0.15, t = 1.73, p = 0.09, 95%CI [-0.04, 0.55]). Additionally, MoCA significantly moderated the relationship between MOFC- MFC connectivity and good versus bad choice (b = 0.10, se = 0.02, t = 4.11, p = 0.006, 95%CI [0.05, 0.15]), showing a significant negative correlation between connectivity and behaviors in low MoCA scores (b = -0.29, se = 0.11, t = -2.74, p = 0.009, 95%CI [-0.50, -0.08]) and a positive correlation in high MoCA scores (b = 0.39, se = 0.13, t = 3.11, p = 0.003, 95%CI [0.14, 0.65]). The FDR was used for correcting multiple comparisons.


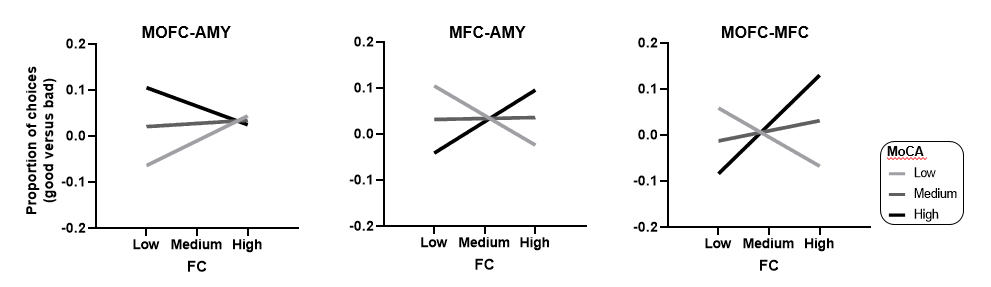

Supplement: Supplementary file 1 — Supplementary file1 (DOCX 206 KB) [file 11357_2023_922_MOESM1_ESM.docx]
